# Identifying *Tmem59 *related gene regulatory network of mouse neural stem cell from a compendium of expression profiles

**DOI:** 10.1186/1752-0509-5-152

**Published:** 2011-09-29

**Authors:** Luwen Zhang, Xiangchun Ju, Yumin Cheng, Xiuyun Guo, Tieqiao Wen

**Affiliations:** 1 Department of Mathematics, College of Science, Shanghai University, 99 Shangda Road, Shanghai 200433, China; 2 Laboratory of Molecular Neurobiology, Institute of Systems Biology, School of Life Sciences, Shanghai University, 99 Shangda Road, Shanghai 200433, China; 3 Shanghai Institute of Applied Mathematics and Mechanics, Shanghai University, 149 Yanchang Road, Shanghai 200072, China

## Abstract

**Background:**

Neural stem cells offer potential treatment for neurodegenerative disorders, such like Alzheimer's disease (AD). While much progress has been made in understanding neural stem cell function, a precise description of the molecular mechanisms regulating neural stem cells is not yet established. This lack of knowledge is a major barrier holding back the discovery of therapeutic uses of neural stem cells. In this paper, the regulatory mechanism of mouse neural stem cell (NSC) differentiation by *tmem59 *is explored on the genome-level.

**Results:**

We identified regulators of *tmem59 *during the differentiation of mouse NSCs from a compendium of expression profiles. Based on the microarray experiment, we developed the parallelized SWNI algorithm to reconstruct gene regulatory networks of mouse neural stem cells. From the inferred *tmem59 *related gene network including 36 genes, *pou6f1 *was identified to regulate *tmem59 *significantly and might play an important role in the differentiation of NSCs in mouse brain. There are four pathways shown in the gene network, indicating that *tmem59 *locates in the downstream of the signalling pathway. The real-time RT-PCR results shown that the over-expression of *pou6f1 *could significantly up-regulate *tmem59 *expression in C17.2 NSC line. 16 out of 36 predicted genes in our constructed network have been reported to be AD-related, including *Ace*, *aqp1*, *arrdc3*, *cd14*, *cd59a*, *cds1*, *cldn1*, *cox8b*, *defb11*, *folr1*, *gdi2*, *mmp3*, *mgp*, *myrip*, *Ripk4*, *rnd3*, and *sncg*. The localization of *tmem59 *related genes and functional-related gene groups based on the Gene Ontology (GO) annotation was also identified.

**Conclusions:**

Our findings suggest that the expression of *tmem59 *is an important factor contributing to AD. The parallelized SWNI algorithm increased the efficiency of network reconstruction significantly. This study enables us to highlight novel genes that may be involved in NSC differentiation and provides a shortcut to identifying genes for AD.

## Background

One of the main goals of systems biology is to determine the biological networks by high performance computing methods and integrating high-throughput data [1,2]. Compared to the traditional biology, which basic strategy is to decypher biological functions by concentrating efforts on a very limited set of molecules, this system-centric approach has an enormous success in producing complex biological networks composed of various types of molecules (genes, proteins, MicroRNAs, etc) from large amounts of data [3].

The microarray technology facilitates large-scale surveys of gene expression data for whole-genome mapping and gene expression analyzing under various conditions [4]. A major focus on microarray data analysis is the reconstruction of gene regulatory networks, which aims to find new gene functions and provide insights into the transcriptional regulation that underlies biological processes [5]. A wide variety of approaches have been proposed to infer gene regulatory networks from microarray data. Those approaches are based on different theories, including Boolean networks [6], Bayesian networks [7], relevance networks [8], graphical models [9], genetic algorithm [10], neural networks [11], controlled language-generating automata [12], linear differential equations [13], and nonlinear differential equations [14]. There are two difficulties that can be addressed for constructing gene networks from gene expression data. Firstly, a single set of gene expression data contains a limited number of time-points under a specific condition. Thus, the problem of determining gene regulatory network becomes an ill-posed one which is difficult to overcome. In the second, while microarray experiments collect an increasing amount of data to be correlated, the network reconstruction is an NP-hard problem. Therefore, application of the statistical framework to a large set of genes requires a prohibitive amount of computing time on a single-CPU. A fundamental problem with the sequential algorithms is their limitation to handle large data sets within a reasonable time and memory resources.

Neurodegenerative disorder, including Alzheimer's disease (AD), Parkinson's disease, and Huntington's diseases etc, is a progressive loss of neurons. Recently, transplantation of NSCs within adult brain has been proposed as one of the potential therapies for neurodegenerative disorders [15]. NSCs are multipotent progenitor cells with long-term, self-renewal and differentiation capabilities to generate three major types of central nervous system (CNS) cell: neurons, astrocytes and oligodendrocytes [16]. They are identified as neuroepithelial cells extending from the ventricle to basal lamina of the pial surface in the initial stage of brain development. During the histogenesis, radial glial stem cells divide asymmetrically to neurons and give rise to astrocytes. Then NSCs become neural progenitorcells existing in the adult brain neurogenic region: the sub-ventricular zone (SVZ) and the sub-granular zone (SGZ) [17-20].

So far the stem cell therapy for neurodegenerative disorders is still a challenging goal [21]. Mechanisms that control the proliferation, differentiation, migration and integration of NSCs are still poorly understood. Comprehensive the gene regulatory network corresponding to NSCs by means of integrating and performing analysis with efficient algorithms is a crucial part of systems biology.

Moreover, mouse transmembrane protein 59 (TMEM59) is an uncharacterized single transmembrane protein. Previously, our study *in vitro *suggested that TMEM59 is differentially expressed during differentiation of primary NSCs from Sprague-Dawley rat striatum [22]. Especially, the down-regulation of TMEM59 with RNAi interference in mouse C17.2 neural stem cell line increases the differentiation of NSCs into neurons and astrocytes [23]. Our study indicated that TMEM59 is related to the differentiation and status sustaining of NSCs. So far the functions of TMEM59 have not yet been reported. Exploration on the tmem59 related gene regulation network of NSCs would help us better understand the molecular mechanism underlying the NSCs differentiation.

In this paper, we constructed gene regulatory networks of mouse NSCs by the parallel strategy on stepwise network inference method. By integrating our microarray data and the public data, the regulatory mechanism of mouse NSCs differentiation by *tmem59 *is explored throughout the genome. The important pathways and the core gene, *pou6f1*, are investigated by Real-time RT-PCR, suggesting that the over-expression of *pou6f1 *significantly up-regulated *tmem59 *expression. We also show that many genes in the *tmem59 *related gene network have been implicated in AD mechanism. The findings enable us to highlight novel genes that may be involved in NSC differentiation and provides a shortcut to identifying genes for AD.

## Methods

### Original data

Microarrays simultaneously quantify thousands of genes on a single glass slide and their use has greatly expanded the breadth of quantified gene expression [24]. In our previous work, six wild and *tmem59 *knockout mice were separately immersed in 75% alcohol for disinfection [25,26]. Under aseptic conditions, the hippocampuses were made into single cell suspension by mechanical whipping. The supernatant was discarded after 900 rmp, 5 min centrifugation. Then the hippocampuses were resuspended in medium (DMEM/F12 culture medium with B27, EGF and bFGF) and were cultured in a glass bottle in CO2 incubator (5% CO2, 37 degree). The gene expression data were measured 4 days later. To understand the biological functions of *tmem59*, we investigated the genes that were differentially expressed due to *tmem59 *knock out. From the *tmem59 *knock out microarray datasets, 627 genes that differentially expressed with more than 2-fold change were selected as our source of data (data not shown).

### Significantly expressed genes selection

In order to focus on much significantly expressed genes related to *tmem59*, we selected 80 genes for further analysis based on the Differential Ratio following *tmem59 *knock out. The precise description of the 80 genes with functions is illustrated in Additional File 1: Table S1.

### Public data selection

In order to examine the regulatory mechanism between *tmem59 *and the corresponding genes, it is necessary to integrate much more microarray data which can be from either in-house or public domain. A good resource for public microarray data is the National Institutes of Health Gene Expression Omnibus http://www.ncbi.nlm.nih.gov/geo/. In this study all the data we used is MIAME compliant and is selected from Gene Expression Omnibus (GEO).

### Microarray data normalization

We transferred the probe data to standard gene expression data. Because a single gene is represented on the array by typically a set of 11-20 pairs of probes, we mapped probes to their corresponding Entrez GeneIDs. Affymetrix probes were mapped to Entrez GeneIDs using the 3 Sep 2010 release of NetAffx annotations. Where probes had multiple GeneID mappings, the one which appears at the top of the GeneID list was selected because been observed that in the majority of such cases the first identifier tends to be the only one with a published symbol as opposed to one that was automatically generated. We calculated the Average Difference for all the probes of the corresponding gene to compare the probe sets expression level of them. The higher the probe set expressed, the larger Average Difference the probes got. Then the expression levels in those probe sets mapped to same gene was summarized. Probe intensities from Affymetrix oligonucleotide microarrays were normalized to gene expression levels using robust multichip analysis (RMA) [27] which is reported to be the single best normalization method compared to MAS5 (Affymetrix), GCRMA, and Dchip PM [28]. The use of ratios or raw intensities is governed by the capabilities of the microarray technology, not by our algorithm.

### Parallelized SWNI Network inference algorithms

We designed and evaluated the Stepwise Network Inference (SWNI) algorithm in previous studies [29]. The SWNI algorithm is a rapid and scalable method of reconstructing gene regulatory networks using gene expression measurements without any prior information about gene functions or network structure. It solves small size problem for high-dimensional data with strict selections in the stepwise regression model. More precisely, the SWNI algorithm infers a module network in two major stages. Firstly, the model is built with ordinary differential equations to describe the dynamics of a gene expression network in perturbation. Secondly, a regression subset-selection strategy is adopted to choose significant regulators for each gene. Moreover, statistical hypothesis testing is used to evaluate the regression model. Then the gene expression network with significant edges and genes is predicted.

However, the SWNI algorithm is a sequential method essentially. While dealing with a large set of genes, the SWNI algorithm requires a prohibitive amount of computing time. To overcome this extreme computational requirement, in this study, we developed a parallel implementation of the SWNI algorithm. Using the message passing interface (MPI), the parallelized SWNI algorithm has higher computing efficiency compared with the SWNI method.

In this study, as same as our own microarray data, the multiple datasets were selected from the experimental platform GPL1261 and were normalized with the RMA algorithm. We subsequently combined all the datasets into a composite training set. The batch adjustment algorithm was applied in the combined training set to ensure that all the datasets were well intermixed [30]. The detail of the parallelized SWNI algorithm is as follows.

A gene expression network is expressed by a set of linear differential equations with each gene expression level as variables, and we have

Ẋ=AX+P,

where *A *= (*a_ij_*)_*n*×*n *_is an *n *× *n *gene regulatory coefficient matrix, and refers to the connectivity of genes in the predictive network; *X *is an *n *× *m *matrix referring to the gene expression level at time *t*; *P *= (*p_ij_*)_*n*×*m *_is a matrix representing the external stimuli (like perturbations) or environment conditions. The computational complexity of the sequential SWNI algorithm is *O*(*n*^3^). In order to reduce the computational complexity, we decomposed *P *by row to partition parallel tasks.

### Assessment of the parallelized SWNI algorithm

Artificial gene networks with random scale-free structure were generated and the distribution of vertices follows a power law. The parallelized SWNI algorithm and the SWNI algorithm have same computing precision. The computing precision of the SWNI algorithm has been discussed in [29]. And the performance of the SWNI algorithm was assessed by comparing the inferred network with the pre-determined artificial network.

The performance of the parallel strategy is evaluated on the artificial gene networks in two important aspects, which are speedup and efficiency. Compared with the SWNI algorithm, the parallelized SWNI algorithm performed better in efficiency. And as the number of processors increases, we got almost linear speedups of the parallelized SWNI algorithm.

### RNA Isolation and Real-time RT-PCR analysis

To study the regulation of *pou6f1 *to *tmem59 *and quantify mRNA by real-time RT-PCR in C17.2 NSCs, we used ReverTra^® ^Ace qPCR RT kit and SYBR^® ^Green Realtime PCR Master Mix (Toyobo Life Science Department).

For Neural stem cell line, C17.2 cells were plated onto 24-well plates at a density of 5 × 10^5 ^cells per well and cultured at 37°C with 5% CO2 for 24 hours before transfection. After reaching about 90% confluence, cells were split. The murine cerebellum-derived immortalized neural stem cell line C17.2 was originally described by Snyder et al. [31].

Full-length cDNA fragment of Pou6f1 was then amplified by RT-PCR using total RNA from mouse brain. The forward primer was 5'-GAAGATCTATGCCCGGGATC AGCAGTC-3' and the reverse primer was 5'-TCCGGAATTCCGGGATCTGAA AGACGTTC-3'. The cDNA was further digested with *Bgl *II/*EcoR *I and subcloned into pEGFP-N2 vector, ultimately sequenced by Invitrogen. The total of 1 ug pEGFP-N2-Pou6f1 DNA per well was used to transfect C17.2 cells using Lipofectamine 2000 at a proportion of 1:1 (according to the manufacturer's protocol). C17.2 cells transfected with pEGFP-N2 in the same condition were used as the control group.

Finally, the total RNA was isolated from each group according to the Trizol manufacture's standard protocol (Takara Bio Inc). PCR primers for amplification of the mouse *tmem59 *gene was specifically design (Invitrogen). Chloroform and isopropanol were used to extract and precipitate the total mRNA. RT-PCR analysis was performed on a PE9700 PCR machine. All reactions were repeated for three times. The relative quantity of *tmem59 *mRNA in the cells was calculated using the equation RQ = 2^-ΔΔCt^. The β-actin was used for normalization as the internal control gene whereas the calibrator was the mean threshold cycle (Ct) value for each control group transfected with pEGFP-N2 vector. The forward primer sequence for *tmem59 *gene is 5'-ATGCTTGTCATCTTGGCTG-3' and the reverse primer sequence is 5'-TCACTTCAGAACG ACCTCA-3'. The forward primer sequence for β-actin is 5'-TGTCCCTGTATGCCT and the reverse primer sequence is 5'-TCACGCACGATTTCCCTC-3'.

### Statistical analysis

Statistical analysis and graph creation were performed by SigmaStat3.5, SigmaPlot 10.0 and Pajek. Data were obtained from at least three independent experiments. Results were presented as means ± SEM. One-way ANOVA was used to analyze the results of real-time PCR. Proportion was analyzed by z-test, and Yates correction was applied to calculations.

## Results

### NSCs related microarrays are selected

We selected microarrays about NSCs, neurogenesis, glias and central nervous system (CNS), due to that NSCs are the principal source of constitutive neurogenesis and glias in the CNS. 146 microarray datasets were selected from 21 different platforms. The species, accession numbers, precise descriptions and number of data sets of the 21 platforms are illustrated in Additional File: Table S2. The comparability of gene expression data generated with different microarray platforms is still a matter of concern. Mixing of data from various platforms could lead to poor results due to quantitative biases among the technologies [32]. Therefore, we selected the datasets including only profiles from a single experimental platform, which ID is identified as GPL1261 in GEO database. In particular, we selected 62 mouse stem cell related sample data sets for further analysis from the Affymetrix Mouse Genome 430 2.0 arrays (Array ([Mouse430_2])), which includes approximately 45, 000 probe sets. The 62 mouse NSC related microarray data sets included in the analysis are illustrated in Table 1.

**Table 1:** 62 mouse neural stem cell related microarray data sets included in the analysis.

Platform: GPL1261			
**Accession**	**Type**	**Samples**	**Description**

**No**.			

GSE12499	NSCs	10	Oct4-Induced Pluripotency in Adult NSCs
GSE10806	Adult NSCs	11	Pluripotent SCs induced from adult NSCs by
			reprogramming with two factors
GSE13379	CNSs	107	Application of a translational profiling approach for the
			comparative analysis of CNS cell types
GSE11862	Neurogenesis	6	Early Gene expression changes after axonal injury
GSE10796	NPCs	4	Identification of genes that restrict astrocyte differentiation
			of midgestational neural precursor cells
GSE11859	NPCs	27	Acquisition of granule neuron precursor identity and
			Hedgehog-induced medulloblastoma in mice
GSE8034	Radial glias	17	Prospective isolation of functionally distinct radial glial
			subtypes - lineage and transcriptome analysis
GSE8091	eNSCs	16	Embryonic brain development
GSE10577	Glia	12	endothelin signaling from photoreceptors to glia
GSE9812	NPCs	22	Molecular heterogeneity of developing retinal ganglion and
			amacrine cells
GSE9763	NPCs	20	Transformed glial progenitor cells
GSE8555	eNSCs	8	D-3-phosphoglycerate dehydrogenase deficiency effect on
			the embryonic head
GSE5817	eNSCs	21	understanding the process of cortical development
GDS2209	CNS	6	Spinal cord and dorsal root ganglion
GDS1017	CNS	15	Hypoxic-ischemic injury response to erythropoietin
			pretreatment
GSE9811	NPCs	42	Individual retinal progenitor cells display extensive
			heterogeneity of gene expression
GSE6675	Astroglia	8	Astroglial gene expression program elicited by
			fibroblast growth factor-2 mande-affy-mouse-307080
GSE5425	CNS	6	Spinal cord and dorsal root ganglion
GSE5011	MSCs	10	molecular changes the MSC acquire through in-vitro
			passages determine their therapeutic potential to EAE
GSE1999	Neurogenesis	15	Hypoxic-ischemic injury response to erythropoietin
			pretreatment
GDS2937	NPCs	6	Olfactory marker protein deficiency effect on the
			olfactory epithelium
GDS2846	Neuron	6	MicroRNA miR-124 expression effect on neuronal cell line
GDS2803	Neurogenesis	4	Fluoxetine effect on the hippocampus
GDS2391	Neurogenesis	6	PGC-1alpha transcriptional coactivator null mutation
GDS2096	Cancer cells	8	Glucocorticoid receptor activation effect on breast cancer
			cells
GDS1793	Neuron	8	Homeodomain interacting protein kinase 2 dominant-negative form effect on trigeminal ganglion
GDS1693	CNS	39	Transcription factor Nrl deficiency effect on photoreceptor
			development
GDS1635	Neuron	20	Nodose and dorsal root ganglia comparison
GDS1084	eNSCs	8	Homeobox Dlx1/2 mutations effect on embryonic
			telencephalon
GSE13386	Neuron	24	Comparative analysis of Drd1+ Medium Spiny Neurons,
			Drd2+ Medium Spiny Neurons, cocaine treatment
GSE13385	Neuron	9	
GSE13384	Neuron	6	
GSE13387	Neuron	24	
GSE13394	CNS	63	A translational profiling approach for the molecular
			characterization of CNS cell types
			characterization of CNS cell types
GSE13379	CNS	107	Application of a translational profiling approach for the
			comparative analysis of CNS cell
GSE11258	Neuron	24	Npas4-regulated genes in mouse hippocampal neurons
GSE10796	NPCs	4	Identification of genes that restrict astrocyte differentiation
			of midgestational neural precursor cells
GSE11859	NPCs	27	Acquisition of granule neuron precursor identity and
			Hedgehog-induced medulloblastoma in mice
GSE8034	Radial glia	17	Prospective isolation of functionally distinct radial glial
			subtypes
GSE11207	Neuron	6	Dorsal root ganglion
GSE11141	NSCs	21	Effects of NgR overexpression on the developing and
			mature forebrain
GSE10360	neuron	2	Role of Endothelin in SCG axon pathfinding
GSE9566	eNSCs	3	PDGF-B induces a homogeneous class of
			oligodendrogliomas from embryonic neural progenitors
GSE9803	Neurogenesis	9	Striatal gene expression data from 12 weeks-old R6/2 mice
			and control mice
GSE9804	Neurogenesis	9	
GSE9330	Neurogenesis	8	Transcription factor Ctip2 deficiency effect on brain striata
GSE6540	Neurogenesis	12	Expression data from olfactory epithelium of Lip-C-treated
			mice compared to Lip-O-treated control mice
GSE9443	CNS	131	Sleep deprivation and the brain
GSE6485	Neurogenesis	6	Expression data from olfactory epithelium of Harlequin
			mutant mice compared to littermate controls
GSE9760	ESC	12	Embryonic stem cells with expanded CAG repeats
GSE6476	Neuron	4	Fluoxetine effect on the hippocampus
GSE8311	Neurogenesis	5	Dlx homeodomain transcription factor mutants
GSE8024	NSC, ESC	8	Murine ES cells, neural precursor cells and embryonic
			fibroblasts
GSE4927	Neurogenesis	6	Olfactory marker protein deficiency effect on the olfactory
			epithelium
GSE6275	CNS	36	neuronal dysfunction associated with the ataxic and epileptic
			phenotypes
GSE4774	Development	15	To determine how Dlx homeobox genes function
GSE4752	CNS	6	target genes regulated by Egr transcriptional regulators
GSE4051	Diff.	8	Photoreceptor-specific nuclear receptor NR2E3 ectopic
			expression effect on NRL null retinas
GSE4041	CNS	6	abnormal expression patterns in NR3B-null mice
GSE2873	Diff.	4	Skeletal muscle synaptic region
GSE2869	Neurogenesis	8	Homeodomain interacting protein kinase 2 dominant-
			negative form effect on trigeminal ganglion
GSE2161	eNSCs	8	Homeobox Dlx1/2 mutations effect on embryonic
			telencephalon

NPCs: Neural progenitor cells; eNSCs: Embryonic neural stem cells; MSCs: Mesenchimal stem cells; ESC: Embryonic stem cells; Diff.: Differentiation.

### The performance of the parallelized SWNI algorithm

**Figure 1:**
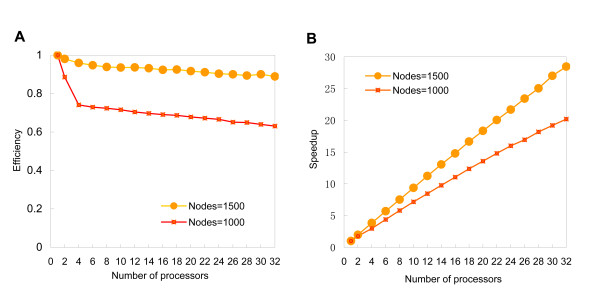
**The efficiency and speedup curves of the parallel SWNI algorithm**. Both of the efficiency and speedup are calculated on two samples. One is an network of 1000 nodes and the other is an network of 1500. (A) Efficiency of the algorithm draw dramatically when processors increased from one to four and then tend to stable. (B) The Speedup is close to a straight line with network of 1500 nodes compared to the network of 1000 nodes.

Following the scale-free topology, we simulated two types of artificial gene networks in size of 1000 nodes, 3054 edges, and 1500 nodes, 4630500 edges, respectively. The performance of the parallelized SWNI algorithm was assessed among the workstation described in the method. Speedup and efficiency of the parallel SWNI algorithm are illustrated in Figure 1, and the running time is shown in Table 2. Figure 1 shows that as the increase of the network scale, the parallelized SWNI algorithm performed better in both efficiency and speedup. Table 2 shows that, as increase in the processor numbers, the computing time of the algorithm falls dramatically. The results demonstrated that the parallelized SWNI algorithm has good performance on the artificial gene networks.

**Table 2:** Computing time of the parallel SWNI algorithm for two types of networks on increased processors.

Number of processors	Network of 1000 nodes	Network of 1500 nodes
1	6501.85	48102.6
2	3670.23	24528.4
4	2193.08	12528.7
6	1485.62	8457.89
8	1122.75	6405.23
10	908.12	5139.61
12	769.32	4280.88
14	666.19	3685.25
16	588.23	3254.62
18	525.86	2887.79
20	479.17	2621.81
22	439.52	2399.3
24	406.73	2217.95
26	383.95	2054
28	357.58	1921.4
30	338.8	1780.58
32	322.09	1689.94

We simulated two types of artificial gene networks in size of 1000 nodes, 3054 edges, and 1500 nodes, 4630500 edges, respectively, to assess the performance of the parallelized SWNI algorithm. The computing time is calculated. The results show that as increase in the processors number, the computing time of the algorithm falls dramatically. The study suggested that the parallelized SWNI algorithm has good performance on the artificial gene networks.

### Gene regulatory networks of mouse neural stem cell

**Figure 2:**
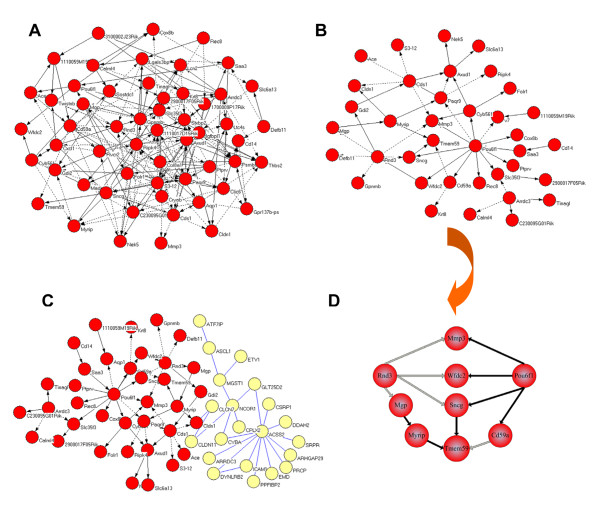
**Predicted gene regulatory networks related to tmem59**. (A) NSC-GN1 is predicted to be a network of 56 genes and 230 edges. (B) NSC-GN2 is predicted to be a network of 37 genes and 46 regulations. (C) NSC-GN3 is identified as a combined network of 39 genes and 29 proteins with 66 regulations and 32 protein-protein interactions. Dark nodes are genes, while light nodes are proteins. (D) NSC-GN4 is extracted from (B) to focus on the precise pathways directed to tmem59 from pou6f1.

GRNs related to *tmem59 *were constructed on a compendium of expression profiles by the parallelized SWNI algorithm (Figure 2). As illustrated in Figure 2A, NSC-GN1 contains 56 genes, 230 edges, and the average degree is 4. From NSC-GN1, *tmem59 *is shown to be negatively regulated by *cd59*, while positively regulated by *sncg*. The global importance of a node in a network can be evaluated by the node degree of it [33]. The basic evaluated strategy is that the bigger the degree of a node is, or the closer to the centre of a network the node is, the more important it is. According to this principle, in NSC-GN1 there are 22 important nodes, which have higher in-degree than the average degree, and can be identified as: *aqp1*, *calml4*, *cd59a*, *clic6*, *cxcl1*, *cyb561*, *flvcr2*, *igfbpl1*, *lgals3bp*, *pou6f1*, *psmb8*, *s3-12*, *sncg arrdc3*, *axud1*, *cds1*, *folr1*, *gpnmb*, *paqr9*, *ptprv*, *ripk4 *and *slc35f3*. Among the 22 nodes, there are 9 more important nodes with twice in-degree than the average degree. Those nodes are *arrdc3*, *axud1*, *cds1*, *folr1*, *gpnmb*, *paqr9*, *ptprv*, *ripk4 *and *slc35f3*.

In order to focus on more significant genes, we rose the significance level of the hypothesis testing in the parallelized SWNI algorithm to delete nodes with lower significant. NSC-GN1 was further extracted to be a sparser one, which is called NSC-GN2 (Figure 2B). It contains nodes and edges with higher positive rate and negative rate compared to nodes and edges in NSC-GN1. 36 genes have significant relationship with *tmem59 *and 46 significant regulatory relationships were identified in NSC-GN2, of which the average node degree is 1.2. *Pou6f1 *regulates 11 genes in NSC-GN2, suggesting that it is the most important gene in it. *Rnd3 *and *cds1 *is related to 5 different genes, respectively. It is worth to mention that, three genes are found to regulate *tmem59*. In the other words, *tmem59 *is negatively regulated by *cd59a*, while positively regulated by *sncg *and *myrip*. Both *cd59a *and *sncg *were also found in NSC-GN1.

Combined with published data, we constructed an integrated network containing both gene regulations and protein-protein interactions with 68 nodes and 98 edges (NSC-GN3 is illustrated in Figure 2C). The average node degree of NSC-GN3 is 1.4. 39 genes, 29 encoded proteins, 66 regulatory relationships and 32 protein-protein interactions are included in NSC-GN3. Partially, gene regulatory relationships of mouse NSCs and differential mechanism of NSCs in protein level is shown in NSC-GN3.

### Novel regulatory pathways

We used the predicted regulatory network of mouse NSCs to infer newly gene interactions. We transformed the location of the nodes in NSC-GN2 and got NSC-GN4 (Figure 2D). From NSC-GN4, four pathways which is related to the expression of *tmeme59 *were obviously identified as

*Pou6f1-Cd59a-Tmem59*,

*Pou6f1-sncg-Tmem59*,

*Pou6f1-Wfdc2-Rnd3-Mgp-Myrip-Tmem59*, and

*Pou6f1-Wfdc2-Rnd3-Sncg-Tmem59*.

All the four pathways initiated from the transcription factor pou6f1. Moreover, the expression of tmem59 is regulated directly by *myrip*, *sncg *and *cd59a*, all of which are regulated by pou6f1 directly or indirectly.

### A novel regulator, *pou6f1*, regulate the expression of *tmem59*

**Figure 3:**
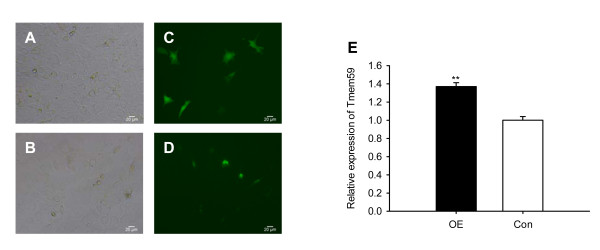
**Positive regulation of pou6f1 to tmem59 is evident in C17.2 NSCs**. (A, B) gray photos were captured in white light. (C, D)POU6F1-EGFP (green) was over-expressed in the nucleus of C17.2 neural stem cells. Photography was captured at 36-hours after transfection of pEGFP-N2-Pou6f1 plasmid. (E) Real-time PCR showed that tmem59 is up-regulated by over-expression of pou6f1 (normalized to β-actin). N = 3, **P < 0.01. OE: over-expression of POU6F1-EGFP; Con: control group transfected with pEGFP-N2.

From Figure 2D, *pou6f1 *is identified to be a dense node, giving hint that *pou6f1 *may play an important role in *tmem59 *expression. In order to confirm this supposition, we constructed an expressional vector to over-express transcription factor POU6F1 fused with EGFP (pEGFP-N2-POU6F1) for real-time observation and quantification in C17.2 NSCs. The results suggested that, POU6F1, a transcription factor, was expressed successfully in the nucleus of NSC compared with ubiquitous location of EGFP (Figure 3A, B, C, D). C17.2 NSCs transfected with pEGFP-N2 vector were used as a control group. Statistically, C17.2 NSCs showed 37.06% ± 4.31% (P < 0.01) increase in *tmem59 *expression caused by the overexpression of *pou6f1 *(Figure 3E). This study firstly identifies a regulator *pou6f1 *that may account for *tmem59 *expression.

### Localization of *tmem59 *related genes and identification of functional-related gene groups

**Figure 4:**
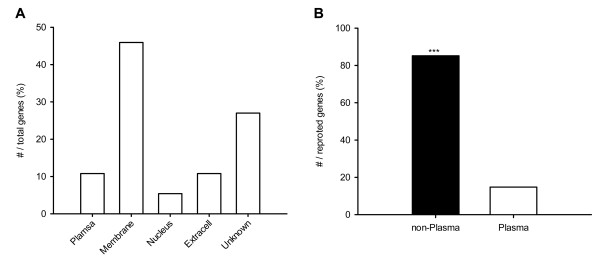
**Location of proteins in Tmem59-related regulatory network**. (A) Distribution of proteins in sub-cellular level. Most of the proteins were located in membrane. (B) Non-Plasma proteins (located in membrane, nucleus and extra-cell) were significantly more than in plasma. Unknown: no notation information in Genebank; ***p < 0.001.

In NSC-GN2 (Figure 2B), 36 genes were predicted to be related to *tmem59 *and 27 of them are annotated in Gene Ontology (GO). Among the 27 annotated proteins, 4, 1, 2 and 4 proteins are localized on plasma, membrane, nucleus and extracellular, respectively. Figure 4 illustrates that 10.8%, 6.0%, 5.4% and 10.8% of all the 37 proteins in NSC-GN2 are localized on different sites, except 27% un-annotated ones.

As mentioned above, the novel membrane proteinTMEM59 modulates complex glycosylation. Based on GO annotation, there are 42% of the 37 proteins involved in metabolism including TMEM59 (Table 3), suggesting that most of the genes have functional similarity with *tmem59*. Beyond that, more than 20% of the 37 proteins are reported to transport materials within cells. The analysis of *tmem59 *related GRN of mouse NSCs highlights new candidate genes involved in (i) peptidase activity, hydrolase activity, kinase activity, and transferase activity; (ii) transportation of water, lipid and metal ion; (iii) protein binding; (iv) transcription process.

**Table 3:** Function of the 37 differentially expressed genes identified in Figure NSC-GN2.

Function	Number of genes	Go accession numbers	Entrez Gene
Enzymatic activity	12	GO:0016787	Ace
		GO:0016740	Cd14
		GO:0016740	Cds1
		GO:0016491	Cox8b
		GO:0005097	Gdi2
		GO:0008237	Mmp3
		GO:0017137	Myrip
		GO:0016301	Nek5
		GO:0016787	Ptprv
		GO:0016301	Ripk4
		GO:0004867	Tmem59
		GO:0004867	Wfdc2
Transporter activity	6	GO:0015250	Aqp1
		GO:0046872	Cyb561
		GO:0008517	Folr1
		GO:0005509	Mgp
		GO:0005319	Saa3
		GO:0005328	Slc6a13
Protein binding	5	GO:0034235	Cd59a
		GO:0016338	Cldn1
		GO:0005178	Gpnmb
		GO:0004872	Paqr9
		GO:0070192	Rec8
Molecular function	4	GO:0003674	Arrdc3
		GO:0003700	Pou6f1
		GO:0000166	Rnd3
		GO:0003674	Sncg
Cytoskeleton	1	GO:0007010	Krt8
Unknown	9	None	1110059m19Rik
		None	2900017f05Rik
		None	Axud1
		None	Calm14
		None	C230095g01Rik
		None	Defb11
		None	S3-12
		None	Slc35f3
		None	Tinag1

Based on the GO annotation, the function of the 37 genes in NSC GN2 is classified. The function, number and Entrez Gene in Genbank are listed as follows. There are 42% proteins having the function of metabolism including TMEM59 in GO-known proteins. Except that, more than 20% proteins function on transporting materials for cells.

### Identification of Alzheimer's disease related genes

It is interesting to address how many genes in *tmem59*-related GRN (NSC-GN2) could be related to Alzheimer's disease (AD). Epigenetic profiling reveals that TMEM59 was down-regulated and lower methylated in major phychosis [34]. And the maturation and localization of amyloid precursor protein (APP) is reported to be modulated by TMEM59 [35]. APP is crucial during the AD pathogenesis, which is often accompanied by some psychotic diseases. In NSC-GN2, *Cd59a*, *myrip *and *sncg *are the three genes which directly regulate *tmem59*, and have been proved to be AD-related in previous reports. In NSC-GN2, our study showed that 17 out of 37 predicted genes (including *tmem59*) are related to AD in NSC-GN2: *Ace *[36], *aqp1 *[37], *arrdc3 *[38], *cd14 *[39], *cd59a *[40], *cds1 *[41], *cldn1 *[42], *cox8b *[43], *defb11 *[44], *folr1 *[45], *gdi2 *[46], *mmp3 *[47], *mgp *[48], *myrip *[49], *Ripk4 *[50], *rnd3 *[51,52], and *sncg *[53]. Among them, *Cd59a*, *myrip *and *sncg *regulate *tmem59 *directly.

## Discussion

*Tmem59 *has been reported to sustain the status of NSCs *in vitro*. Knockout of *tmem59 *in mouse brain can induce expressional changes of 627 genes in neonatal mouse NSCs. Until now, the underlying function of *tmem59*, especially on the differentiation of mouse NSCs, is still unclear. In this study, we try to find out regulators likely to affect the gene expression in mouse NSC and new mechanism of neurodegeneration in AD from a compendium of expression profiles.

Firstly, 36 genes were identified to be *tmem59 *related. In the predicted network NSC-GN2, *tmem59 *is regulated directly by *cd59a*, *myrip *and *sncg*. Meanwhile, four pathways were found in NSC-GN2 to regulate the expression of *tmem59 *from *pou6f1*. *Tmem59 *is located downstream in all the pathways, indicating that *tmem59 *is probably regulated by all the other genes. These conclusions are in accordance with observations from earlier studies [23]. Our study suggests that the 36 genes probably act on the differentiation of NSCs and have similar function with *tmem59*.

Secondly, Our RT PCR analysis results shown that *tmem59 *is positively regulated by *pou6f1*. And *pou6f1 *has been reported to play an important role during the development of mouse telencephalon [54]. Our study suggests that the influence of *pou6f1 *on mouse telencephalon development is originated from the effect on NSCs during the mouse embryonic development. This study provides further insights into the role of the differentiation of NSCs.

Thirdly, our study suggests that TMEM59 has similar localization with most of its regulators. Recently, TMEM59 was reported to be a Golgi-localized protein, which is crucial in modulated complex glycosylation, cell surface expression and secretion of amyloid precursor protein [34]. As known, proteins in the cell plasma are synthesized directly in free ribosome, while some other membrane proteins which transfer to the nucleus, are synthesized in rough endoplasmic reticulum. The second type of protein will be transported to subcellular location secreted by Golgi-complex. Among the 27 annotated genes in the predicted network NSC-GN2, more than 85% were identified to be nonplasmic localized. This suggests that 85% of the 27 proteins are Golgi-localized in maturation and has similar localization with TMEM59.

Furthermore, our study suggests that the *tmem59 *related gene regulatory network (NSC-GN2) is probably AD-related. As the precursor of β-amyloid protein (Aβ), β-amyloid precursor protein (APP) is addressed to be the first genetic mutation. The deposition of Aβ in plaques of brain is already identified to be the cause of AD. As been reported, TMEM59 is Golgi-localized in Hek293 cell line, and modulate the complex glycosylation, cell surface expression and secretion of APP. The study indicates that TMEM59 may be associated with AD. In our predicted mouse NSCs related network NSC-GN2, three genes which regulate *Tmem59 *directly are identified as *sncg*, *cd59a *and *myrip*. *Sncg *(γ-synuclein) has been identified to be correlated to dementia hippocampus of AD and pathology of Parkinson's disease (PD) [55]. Deficiency of complement regulator *cd59a *is the cause of neurodegeneration in AD [56]. And *Rab27 *binding protein MYRIP is involved in insulin exocytosis, impaired which is the pathogenesis of AD [57,58]. Besides, there are nearly 50% of all the genes in NSC-GN2 have been reported to be directly or indirectly related to AD. Therefore, *tmem59*, which directly regulated by *cd59a*, *myrip *and *sncg*is, is suggested to be associated with AD, and the unreported genes in NSC-GN2 are probably related to AD either.

## Conclusions

In this study, we predicted the mouse NSCs related GRNs by the parallelized SWNI algorithm integrating data from the *tmem59 *knock out microarray datasets and 62 mouse stem cell related microarray datasets in GEO. The parallelized SWNI algorithm increased the efficiency of network reconstruction significantly. In particular, a high confident network of mouse NSCs (NSC-GN2) was predicted. In the network, 36 key genes regulating *tmem59 *expression were identified. The RT PCR result suggested that *tmem59 *can be positively regulated by *pou6f1 *significantly. Moreover, 17 out of 36 genes are predicted to be AD related in our network including *tmem59*. This is in coherence with published references.

This present work provides new insights regarding the gene regulations of NSCs. The parallel methods presented in this paper might also become a scalable tool for large-scale analysis on various types of cells and species. And integration of multiple datasets will provide for new research directions in microarray analysis. This study enables us to highlight novel genes that may be involved in NSC differentiation and provides a shortcut to identify genes for AD.

## Authors' contributions

LWZ designed and implemented the methods and wrote the initial draft of the manuscript. XCJ conducted the experiments and contributed to the analyses. YMC performed the method and conceived the study. XYG derived the mathematical model and added some new features. TQW provided the biological insight to the manuscript and contributed to the final manuscript. All the authors read, edited and approved the final manuscript.

## Supplementary Material

Additional file 1 **Table S1 for 80 selected genes lists from the tmem59 knock-out microarray experiment included in the analysis**. From the tmem59 knock out microarray datasets, 627 genes that differentially expressed with more than 2-fold change were selected as our source of data. In order to focus on much significantly expressed genes related to tmem59, we selected 80 genes for further analysis based on the Differential Ratio following tmem59 knock out. The symbol, Gene ID and function of each gene can be searched in Genbank. Click here for file

Additional file 2 **Table S2 for 21 platforms related to 146 microarray datasets about mouse NSCs**. Microarrays about NSCs, neurogenesis, glias and central nervous system (CNS) are selected, due to that NSCs are the principal source of constitutive neurogenesis and glias in the CNS. 146 microarray datasets were selected from 21 different platforms for constructing genes regulatory network of mouse NSC. The species, accession numbers, precise descriptions and number of data sets of the 21 platforms are illustrated. Click here for file
