# Supplementary material for: Identifying Tmem59 related gene regulatory network of mouse neural stem cell from a compendium of expression profiles
Source: BMC Syst Biol. 2011 Sep 29;5:152. doi: 10.1186/1752-0509-5-152 (PMC3191490; doi:10.1186/1752-0509-5-152)
Supplement: Additional file 1 — Table S1 for 80 selected genes lists from the tmem59 knock-out microarray experiment included in the analysis. From the tmem59 knock out microarray datasets, 627 genes that differentially expressed with more than 2-fold change were selected as our source of data. In order to focus on much significantly expressed genes related to tmem59, we selected 80 genes for further analysis based on the Differential Ratio following tmem59 knock out. The symbol, Gene ID and function of each gene can be searched in Genbank. [file 1752-0509-5-152-S1.DOC]

# 0BTable S1. 80 selected genes lists from the tmem59 knock-out microarray experiment

| **Symbol** | **Gene ID** | **Function** | **Reference** |
| --- | --- | --- | --- |
| 1110017D15Rik | 73721 | Cellular protein complex assembly | [1] |
| 1110059M19Rik | 68800 |  |  |
| 1700009P17Rik | 75472 |  |  |
| 2410146L05Rik | 67968 | Cellular protein complex assembly; embryonic | [2], [3] |
|  |  | pattern specification, protein phosphorylation |  |
| 2610304F08Rik | 278507 | Transforming growth factor beta receptor | [4] |
|  |  | signaling pathway |  |
| 2900017F05Rik | 72915 |  |  |
| 2900040C04Rik | 72893 |  |  |
| 3100002J23Rik | 75429 |  |  |
| 5330423N11Rik | N/A |  |  |
| 6430530L21Rik | N/A |  |  |
| 8430438L13Rik | 234396 |  |  |
| 9630013P03Rik | N/A |  |  |
| 9630038C08Rik | N/A |  |  |
| Ace | 11421 | peptidyl-dipeptidase and metallopeptidase activity; neutrophil mediated immnity | [5], [6] |
|  |  | activity; neutrophil mediated immnity |  |
| AI451557 | 434341 |  |  |
| Aqp1 | 11826 | KP^+P^ and HR_2R_O transmembrane transporter | [7], [8], [9] |
|  |  | activity; camera-type eye morphogenesis |  |
| Arrdc3 | 105171 |  |  |
| Axud1 | 215418 | Transcription factor; post-embryonic | [10], [11] |
|  |  | development |  |
| B230312I18Rik | 233058 |  |  |
| Bst2 | 69550 |  |  |
| C230053E11Rik | 110333 |  |  |
| C230095G01Rik | 319776 |  |  |
| C730043O17 | 384198 |  |  |
| Calml4 | 75600 |  |  |
| Cd14 | 12475 |  |  |
| Cd59a | 12509 |  |  |
| Cds1 | 74596 |  |  |
| Cldn1 | 12737 | Protein binding | [12], [13] |
| Clic6 | 209195 |  |  |
| Col8a1 | 12837 | Positive regulation of cell-substrate adhesion, | [14], [15] |
|  |  | ephithelial cell proliferation |  |
| Cox8b | 12869 |  |  |
| Cryab | 12955 | Apoptosis involved in morphogenesis; camera- | [16], [17] |
|  |  | type eye development |  |
| Cxcl1 | 14825 |  |  |
| Cyb561 | 13056 | Ferric-chelate reductase activity | [18] |
| D130086K05Rik | 319758 |  |  |
| D230046H12Rik | N/A |  |  |
| D630035D13Rik | N/A |  |  |
| D630047N04Rik | N/A |  |  |
| Defb11 | 246081 |  |  |
| F730021A14Rik | N/A |  |  |
| Flvcr2 | 217721 |  |  |
| Folr1 | 14275 | Folic acid mebabolism | [19] |
| Gdi2 | 14569 | Small GTPase mediated signal transduction | [20] |
| Gpnmb | 93695 | Cell adhesion | [21] |
| Gpr137b-ps | 664862 |  |  |
| Igfbp5 | 16011 | Regulation of cell growth | [22] |
| Krt8 | 16691 | Cell morphogenesis, apoptosis, tumor necrosis | [23], [24] |
|  |  | Factor-mediated signaling |  |
| Lcn2 | 16819 | Apoptosis, innate immune response | [25], [26] |
| Lgals3bp | 19039 | Signal transduction | [27] |
| LOC100047583 | 100047583 |  |  |
| LOC100048733 | 100048733 |  |  |
| LOC641366 | 641366 |  |  |
| LOC674912 | 674912 |  |  |
| LOC675572 | 675572 |  |  |
| Ltc4s | 17001 | Leukotriene metabolism | [28] |
| Madcam1 | 17123 | Keratinocyte differentiation | [29] |
| Mgp | 17313 |  |  |
| Mmp3 | 17392 |  |  |
| Myrip | 245049 | Regulation of catalytic activity, vesicle | [30], [31] |
|  |  | transporte along actin filament |  |
| Nek5 | 330721 |  |  |
| P | 18431 | Cell proliferation; pigmentation; melanocyte | [32], [33], [34] |
|  |  | differentiation |  |
| Paqr9 | 75552 |  |  |
| Pou6f1 | 19009 | Transcription factor | [35] |
| Psmb8 | 16913 | Antigen processing and presentation | [36] |
| Ptprv | 13924 | Apoptosis, DNA damage response, protein | [37], [38], [37] |
|  |  | carboxylation |  |
| Rec8 | 56739 | Meiosis, oocyte maturation; spermatid | [39], [40], [40] |
|  |  | development |  |
| Ripk4 | 72388 |  |  |
| Rnd3 | 74194 |  |  |
| S3-12 | 57435 |  |  |
| Saa3 | 20210 |  |  |
| Slc35f3 | 210027 |  |  |
| Slc6a13 | 14412 |  |  |
| Sncg | 20618 | Dopamine secretion | [41] |
| Sostdc1 | 66042 | Regulation of BMP signaling pathway; pattern | [42], [43] |
|  |  | specification process |  |
| Stxbp2 | 20911 | Apical plasma membrane | [44] |
| Thbs2 | 21826 |  |  |
| Tinagl | 94242 | Laminin binding | [45] |
| Tmem59 | 56374 |  |  |
| Twistnb | 28071 |  |  |
| Wfdc2 | 67701 |  |  |

From the tmem59 knock out microarray datasets, 627 genes that differentially expressed with more than 2-fold change were selected as our source of data. In order to focus on much significantly expressed genes related to tmem59, we selected 80 genes for further analysis based on the Differential Ratio following tmem59 knock out. The symbol, Gene ID and function of each gene can be searched in Genbank.

## References:

1. Matsuoka Y, Miyagawa Y, Tokuhiro K, Kitamura K, Iguchi N, et al.: **Isolation and characterization of the spermatid-specific Smrp1 gene encoding a novel manchette protein.** *Mol Reprod Dev* 2008, **75**: 967-975.
2. Herr JC, Chertihin O, Digilio L, Jha KN, Vemuganti S, et al.: **Distribution of RNA binding protein MOEP19 in the oocyte cortex and early embryo indicates pre-patterning related to blastomere polarity and trophectoderm specification.** *Dev Biol* 2008, **314**: 300-316.
3. Li L, Baibakov B, Dean J: **A subcortical maternal complex essential for preimplantation mouse embryogenesis.** *Dev Cell* 2008, **15**: 416-425.
4. Hill JJ, Qiu Y, Hewick RM, Wolfman NM: **Regulation of myostatin in vivo by growth and differentiation factor-associated serum protein-1: a novel protein with protease inhibitor and follistatin domains.** *Mol Endocrinol* 2003, **17**: 1144-1154.
5. Junot C, Gonzales MF, Ezan E, Cotton J, Vazeux G, et al.: **RXP 407, a selective inhibitor of the N-domain of angiotensin I-converting enzyme, blocks in vivo the degradation of hemoregulatory peptide acetyl-Ser-Asp-Lys-Pro with no effect on angiotensin I hydrolysis.** *J Pharmacol Exp Ther* 2010, **297**: 606-611.
6. Arndt PG, Young SK, Poch KR, Nick JA, Falk S, et al.: **Systemic inhibition of the angiotensin-converting enzyme limits lipopolysaccharide-induced lung neutrophil recruitment through both bradykinin and angiotensin II-regulated pathways.** *J Immunol* 2006, **177**: 7233-7241.
7. Radtke AL, O'Riordan MX: **Homeostatic maintenance of pathogen-containing vacuoles requires TBK1-dependent regulation of aquaporin-1.** *Cell Microbiol* 2008, **10**: 2197-2207.
8. Offenberg H, Barcroft LC, Caveney A, Viuff D, Thomsen PD, et al.: **mRNAs encoding aquaporins are present during murine preimplantation development.** *Mol Reprod Dev* 2000, **57**: 323-330.
9. Thiagarajah JR, Verkman AS: **Aquaporin deletion in mice reduces corneal water permeability and delays restoration of transparency after swelling.** J *Biol Chem* 2002, **277**: 19139-19144.
10. Gingras S, Pelletier S, Boyd K, Ihle JN: **Characterization of a family of novel cysteine- serine-rich nuclear proteins (CSRNP).** *PLoS One* 2007, **2**: e808.
11. Schmahl J, Raymond CS, Soriano P: **PDGF signaling specificity is mediated through multiple immediate early genes.** *Nat Genet* 2007, **39**: 52-60.
12. Hamazaki Y, Itoh M, Sasaki H, Furuse M, Tsukita S: **Multi-PDZ domain protein 1 (MUPP1) is concentrated at tight junctions through its possible interaction with claudin-1 and junctional adhesion molecule.** *J Biol Chem* 2002, **277**: 455-461.
13. Furuse M, Sasaki H, Tsukita S: **Manner of interaction of heterogeneous claudin species within and between tight junction strands**. *J Cell Biol* 1999, **147**: 891-903.
14. Hopfer U, Fukai N, Hopfer H, Wolf G, Joyce N, et al.: **Targeted disruption of Col8a1 and Col8a2 genes in mice leads to anterior segment abnormalities in the eye.** *FASEB J* 2005, **19**: 1232-1244.
15. Manabe R, Tsutsui K, Yamada T, Kimura M, Nakano I, et al.: Transcriptome-based systematic identification of extracellular matrix proteins. Proc Natl Acad Sci U S A 105: 12849-12854.
16. Morozov V, Wawrousek EF: **Caspase-dependent secondary lens fiber cell disintegration in alphaA-/alphaB-crystallin double-knockout mice.** *Development* 2006, **133**: 813-821.
17. Boyle DL, Takemoto L, Brady JP, Wawrousek EF: **Morphological characterization of the Alpha A- and Alpha B-crystallin double knockout mouse lens.** *BMC Ophthalmol* 2003, **3**: 3.
18. Vargas JD, Herpers B, McKie AT, Gledhill S, McDonnell J, et al.: **Stromal cell-derived receptor 2 and cytochrome b561 are functional ferric reductases.** *Biochim Biophys Acta* 2003, **1651**: 116-123.
19. Tang LS, Finnell RH: **Neural and orofacial defects in Folp1 knockout mice.** *Birth Defects Res A Clin Mol Teratol* 2003, **67**: 209-218.
20. Janoueix-Lerosey I, Jollivet F, Camonis J, Marche PN, Goud B: **Two-hybrid system screen with the small GTP-binding protein Rab6. Identification of a novel mouse GDP dissociation inhibitor isoform and two other potential partners of Rab6.** *J Biol Chem* 1995, **270**: 14801-14808.
21. Shikano S, Bonkobara M, Zukas PK, Ariizumi K: **Molecular cloning of a dendritic cell-associated transmembrane protein, DC-HIL, that promotes RGD-dependent adhesion of endothelial cells through recognition of heparan sulfate proteoglycans.** *J Biol Chem* 2001, **276**: 8125-8134.
22. McCusker RH, Novakofski J: **Zinc partitions insulin-like growth factors (IGFs) from soluble IGF binding protein (IGFBP)-5 to the cell surface receptors of BC3H-1 muscle cells.** *J Cell Physiol* 2003, **197**: 388-399.
23. Caulin C, Ware CF, Magin TM, Oshima RG: **Keratin-dependent, epithelial resistance to tumor necrosis factor-induced apoptosis.** *J Cell Biol* 2000, **149**: 17-22.
24. Ouellet T, Levac P, Royal A: **Complete sequence of the mouse type-II keratin EndoA: its amino-terminal region resembles mitochondrial signal peptides.** **Gene** 1988, *70*: 75-84.
25. Berger T, Togawa A, Duncan GS, Elia AJ, You-Ten A, et al.: **Lipocalin 2-deficient mice exhibit increased sensitivity to Escherichia coli infection but not to ischemia-reperfusion injury.** *Proc Natl Acad Sci* 2006, **103**: 1834-1839.
26. Devireddy LR, Gazin C, Zhu X, Green MR: **A cell-surface receptor for lipocalin 24p3 selectively mediates apoptosis and iron uptake.** *Cell* 2005, **123**: 1293-1305.
27. Chicheportiche Y, Vassalli P: **Cloning and expression of a mouse macrophage cDNA coding for a membrane glycoprotein of the scavenger receptor cysteine-rich domain family.** *J Biol Chem* 1994, **269**: 5512-5517.
28. Lam BK, Penrose JF, Rokach J, Xu K, Baldasaro MH, et al.: **Molecular cloning, expression and characterization of mouse leukotriene C4 synthase.** *Eur J Biochem* 1996, **238**: 606-612.
29. Nishioka E, Tanaka T, Yoshida H, Matsumura K, Nishikawa S, et al.: **Mucosal addressin cell adhesion molecule 1 plays an unexpected role in the development of mouse guard hair.** *J Invest Dermatol* 2002, **119**: 632-638.
30. El-Amraoui A, Schonn JS, Kussel-Andermann P, Blanchard S, Desnos C, et al.: **MyRIP, a novel Rab effector, enables myosin VIIa recruitment to retinal melanosomes.** *EMBO Rep* 2002, **3**: 463-470.
31. Fukuda M, Kuroda TS: **Slac2-c (synaptotagmin-like protein homologue lacking C2 domains-c), a novel linker protein that interacts with Rab27, myosin Va/VIIa, and actin.** *J Biol Chem* 2002, **277**: 43096-43103.
32. Hirobe T, Wakamatsu K, Ito S: **Effects of genic substitution at the agouti, brown, albino, dilute, and pink-eyed dilution loci on the proliferation and differentiation of mouse epidermal melanocytes in serum-free culture.** *Eur J Cell Biol* 1998, **75**: 184-191.
33. Moore KJ, Swing DA, Copeland NG, Jenkins NA: **Interaction of the murine dilute suppressor gene (dsu) with fourteen coat color mutations.** *Genetics* 1990, **125**: 421-430.
34. Markert CL, Silvers WK: **The Effects of Genotype and Cell Environment on Melanoblast Differentiation in the House Mouse.** *Genetics* 1956, **41**: 429-450.
35. Okamoto K, Wakamiya M, Noji S, Koyama E, Taniguchi S, et al.: **A novel class of murine POU gene predominantly expressed in central nervous system.** *J Biol Chem* 1993, **268**: 7449-7457.
36. Basler M, Youhnovski N, Van Den Broek M, Przybylski M, Groettrup M: **Immunoproteasomes down-regulate presentation of a subdominant T cell epitope from lymphocytic choriomeningitis virus.** *J Immunol* 2004, **173**: 3925-3934.
37. Doumont G, Martoriati A, Beekman C, Bogaerts S, Mee PJ, et al.: **G1 checkpoint failure and increased tumor susceptibility in mice lacking the novel p53 target Ptprv.** *EMBO J* 2005, **24**: 3093-3103.
38. Lee NK, Sowa H, Hinoi E, Ferron M, Ahn JD, et al.: **Endocrine regulation of energy metabolism by the skeleton.** *Cell* 2007, **130**: 456-469.
39. Lee J, Iwai T, Yokota T, Yamashita M: **Temporally and spatially selective loss of Rec8 protein from meiotic chromosomes during mammalian meiosis.** *J Cell Sci* 2003, **116**: 2781-2790.
40. Li XC, Schimenti JC: **Mouse pachytene checkpoint 2 (trip13) is required for completing meiotic recombination but not synapsis.** *PLoS Genet* 2007, **3**: e130.
41. Senior SL, Ninkina N, Deacon R, Bannerman D, Buchman VL, et al.: **Increased striatal dopamine release and hyperdopaminergic-like behaviour in mice lacking both alpha-synuclein and gamma-synuclein.** *Eur J Neurosci* 2008, **27**: 947-957.
42. Laurikkala J, Kassai Y, Pakkasjarvi L, Thesleff I, Itoh N: **Identification of a secreted BMP antagonist, ectodin, integrating BMP, FGF, and SHH signals from the tooth enamel knot.** *Dev Biol* 2003, **264**: 91-105.
43. Kassai Y, Munne P, Hotta Y, Penttila E, Kavanagh K, et al.: **Regulation of mammalian tooth cusp patterning by ectodin.** *Science* 2005, **309**: 2067-2070.
44. Procino G, Barbieri C, Tamma G, De Benedictis L, Pessin JE, et al.: **AQP2 exocytosis in the renal collecting duct -- involvement of SNARE isoforms and the regulatory role of Munc18b.** *J Cell Sci* 2008, **121**: 2097-2106.
45. Igarashi T, Tajiri Y, Sakurai M, Sato E, Li D, et al.: **Tubulointerstitial nephritis antigen-like 1 is expressed in extraembryonic tissues and interacts with laminin 1 in the Reichert membrane at postimplantation in the mouse.** *Biol Repord* 2009, **81**: 948-955.
