# Supplementary material for: Identifying Tmem59 related gene regulatory network of mouse neural stem cell from a compendium of expression profiles
Source: BMC Syst Biol. 2011 Sep 29;5:152. doi: 10.1186/1752-0509-5-152 (PMC3191490; doi:10.1186/1752-0509-5-152)
Supplement: Additional file 2 — Table S2 for 21 platforms related to 146 microarray datasets about mouse NSCs. Microarrays about NSCs, neurogenesis, glias and central nervous system (CNS) are selected, due to that NSCs are the principal source of constitutive neurogenesis and glias in the CNS. 146 microarray datasets were selected from 21 different platforms for constructing genes regulatory network of mouse NSC. The species, accession numbers, precise descriptions and number of data sets of the 21 platforms are illustrated. [file 1752-0509-5-152-S2.DOC]

## Table S2. 21 platforms related to 146 microarray datasets about mouse NSCs.

| **Species** | **Accession No.** | **Platform description** | **No. of data sets** |
| --- | --- | --- | --- |
| mouse | GPL1261 | Affymetrix Mouse Genome 430 2.0 Array | 62 |
| mouse | GPL1349 | SAGE:17:NlaIII:Mus musculus | 1 |
| mouse | GPL2872 | Agilent-012694 Whole Mouse Genome G4122A | 9 |
| mouse | GPL2987 | Macrogen MAC Mouse 11K Oligo Microarray | 1 |
| mouse | GPL32 | Affymetrix Murine Genome U74A Array | 2 |
| mouse | GPL339 | Affymetrix Mouse Expression 430A Array | 22 |
| mouse | GPL340 | Affymetrix Mouse Expression 430B Array | 2 |
| mouse | GPL3571 | custom mouse IMAGE GEM1 8.734K | 1 |
| mouse | GPL6096 | Affymetrix Mouse Exon 1.0 ST Array | 3 |
| mouse | GPL6100 | Illumina mouseRef-8 v1.0 expression beadchip | 1 |
| mouse | GPL6103 | Illumina mouseRef-8 v1.1 expression beadchip | 1 |
| mouse | GPL6126 | ENS SGDB Mus musculus NIA 15k | 1 |
| mouse | GPL6145 | ENS SGDB Mus musculus NeuroDev v2.2 | 1 |
| mouse | GPL6231 | VMSR Mus musculus MEEBO 38.5K v1.0 | 1 |
| mouse | GPL7877 | BRC Mus musculus 3K Feb 23, 2009 | 1 |
| mouse | GPL81 | Affymetrix Murine Genome U74 Version 2 Array | 23 |
| mouse | GPL82 | Affymetrix Murine Genome U74 Version 2 Array | 4 |
| mouse | GPL8229 | PGA Mouse v1.1 | 1 |
| mouse | GPL83 | Affymetrix Murine Genome U74 Version 2 Array | 2 |
| mouse | GPL8321 | Affymetrix Mouse Genome 430A 2.0 Array | 6 |

Microarrays about NSCs, neurogenesis, glias and central nervous system (CNS) are selected, due to that NSCs are the principal source of constitutive neurogenesis and glias in the CNS. 146 microarray datasets were selected from 21 different platforms for constructing genes regulatory network of mouse NSC. The species, accession numbers, precise descriptions and number of data sets of the 21 platforms are illustrated.
